# Endogenous SIRT6 in platelets negatively regulates platelet activation and thrombosis

**DOI:** 10.3389/fphar.2023.1268708

**Published:** 2023-12-18

**Authors:** Yanli Liu, Tao Wang, Qilong Zhou, Guang Xin, Hai Niu, Fan Li, Yilan Wang, Shiyi Li, Yuman Dong, Kun Zhang, Lijuan Feng, Wei Fu, Boli Zhang, Wen Huang

**Affiliations:** ^1^ Department of Neurosurgery, Laboratory of Ethnopharmacology, Tissue-orientated Property of Chinese Medicine Key Laboratory of Sichuan Province, West China School of Medicine, West China Hospital, Sichuan University, Chengdu, China; ^2^ Innovative Chinese Medicine Academician Workstation, West China Hospital, Sichuan University, Chengdu, China

**Keywords:** platelet activation, cardiovascular-related disease, SIRT6, thrombosis, MAPK

## Abstract

Thromboembolism resulting from platelet dysfunction constitutes a significant contributor to the development of cardiovascular disease. Sirtuin 6 (SIRT6), an essential NAD^+^-dependent enzyme, has been linked to arterial thrombosis when absent in endothelial cells. In the present study, we have confirmed the presence of SIRT6 protein in anucleated platelets. However, the precise regulatory role of platelet endogenous SIRT6 in platelet activation and thrombotic processes has remained uncertain. Herein, we present compelling evidence demonstrating that platelets isolated from SIRT6-knockout mice (SIRT6^−/−^) exhibit a notable augmentation in thrombin-induced platelet activation, aggregation, and clot retraction. In contrast, activation of SIRT6 through specific agonist treatment (UBCS039) confers a pronounced protective effect on platelet activation and arterial thrombosis. Moreover, in platelet adoptive transfer experiments between wild-type (WT) and SIRT6^−/−^ mice, the loss of SIRT6 in platelets significantly prolongs the mean thrombus occlusion time in a FeCl_3_-induced arterial thrombosis mouse model. Mechanistically, we have identified that SIRT6 deficiency in platelets leads to the enhanced expression and release of proprotein convertase subtilisin/kexin type 9 (PCSK9), subsequently activating the platelet activation-associated mitogen-activated protein kinase (MAPK) signaling pathway. These findings collectively unveil a novel protective role of platelet endogenous SIRT6 in platelet activation and thrombosis. This protective effect is, at least in part, attributed to the inhibition of platelet PCSK9 secretion and mitogen-activated protein kinase signaling transduction. Our study provides valuable insights into the intricate interplay between SIRT6 and platelet function, shedding light on potential therapeutic avenues for managing thrombotic disorders.

## 1 Introduction

Thromboembolism constitutes a prominent underlying factor in the development of cardiovascular diseases (Furie and Furie, 2008; Koupenova et al., 2017). The prevalence of thrombus-related incidents is alarmingly high, accounting for nearly a quarter of global mortality rates annually (Massussi et al., 2021). Currently, antiplatelet drugs are widely used for the clinical prevention and treatment of thrombosis (Ridker et al., 2005; Paniccia et al., 2015; Kalmanti and Lindhoff-Last, 2020), however, a notable challenge arises from the fact that approximately one-third of thrombotic patients exhibit biochemical resistance to these antiplatelet-activating medications. Therefore, gaining a precise understanding of the mechanisms underlying platelet activation holds immense significance for the development of new antiplatelet drugs.

Sirtuins (SIRT) are a class of NAD^+^-dependent protein deacylases and ADP-ribosyl-transferases exert protective roles in cell cycle, metabolic regulation, apoptosis, inflammation and oxidative stress (D'Onofrio et al., 2015; Grootaert and Bennett, 2022). Early studies have revealed that members of the sirtuin family, such as SIRT1 and SIRT3, play important protective roles in arterial thrombosis (Breitenstein et al., 2011; Gaul et al., 2018). Specifically, inhibition of SIRT1 has been shown to enhance tissue factor (TF) expression and activity through activation of the NF-κB signaling pathway, thereby exacerbating arterial thrombosis (Breitenstein et al., 2011); and SIRT3 deficiency in mice resulted in a marked enhancement of carotid artery occlusion via increased neutrophil extracellular traps and elevated plasma TF levels (Gaul et al., 2018). Considering that SIRT family members have a highly conserved catalytic core, other members of the SIRT family may also be involved in the regulation of thrombus formation.

SIRT6, a well-studied SIRT family members, plays a critical role in human diseases like cancer, diabetes, inflammation and cardiovascular diseases (Huang et al., 2018; Tian et al., 2019; Liu et al., 2021). Interestingly, SIRT6 has been found to inhibit the secretion of proprotein convertase subtilisin/kexin type 9 (PCSK9) (Tao et al., 2013). Given that PCSK9 can induce platelet activation by binding to CD36 receptors on platelets (Qi et al., 2021), this suggests that SIRT6 may exert a regulatory effect on platelet function. Coincidentally, a recent study demonstrated that endothelium-specific SIRT6 deletion promotes arterial thrombosis in mice by enhancing TF expression and activating pro-inflammatory signaling (Gaul et al., 2023). In view of the pivotal role of platelets in thrombosis, it may be of great significance to explore whether endogenous SIRT6 in platelets also plays a regulatory role in thrombotic processes.

In this study, we demonstrate that SIRT6 is expressed in mouse platelets and negatively regulate thrombin-induced platelet activation and FeCl_3_-induced arterial thrombosis in mice. We have also identified that SIRT6 exerts its inhibitory influence on platelet activation primarily by suppressing PCSK9 expression and secretion, consequently leading to the downregulation of the mitogen-activated protein kinase (MAPK) signaling pathway. These findings offer valuable contributions to our comprehensive understanding of how SIRT6 precisely regulates platelet function and the intricate process of thrombus formation.

## 2 Materials and methods

### 2.1 Materials

UBCS039 was procured from MedChemExpress (HY-115453, NJ, United States). Fibrinogen (341,576) and thrombin (T7513) were obtained from Sigma-Aldrich (MA, United States). Protein concentration was determined using a BCA protein assay kit (P0012, Beyotime Biotechnology, Shanghai, CN). The following primary antibodies were utilized: anti-phospho-p38 (#9211), anti-p38 (#9212), anti-phospho-JNK (#9252), anti-JNK (#9251), anti-phospho-ERK (#9101), and anti-ERK (#9102), all sourced from Cell Signaling Technology (MA, United States) at a dilution of 1:1000. The secondary antibodies, goat anti-rabbit (A0208) and goat anti-mouse (A0216), were purchased from Beyotime Biotechnology (Shanghai, CN). PE-CD62P (148,036) and FITC-integrin αIIbβ3 (362,803) were procured from BioLegend (CA, United States). The Anti-CD42b antibody (R300) was purchased from Emfret Analytics (Würzburg, Germany).

### 2.2 Mice

SPF male C57BL/6 mice (Chengdu Dashuo Experimental Animal Co., Ltd., license number: SCXKchuan 2015-030) weighing 23 ± 2 g were used, and the Ethics Committee of the West China Hospital of Sichuan University approved all experiments. SIRT6 knockout (SIRT6^−/−^) mice on C57BL/6J background were donated by Dr Zhang, Dental Research Institute of West China Hospital. The Mice Gene Identification Sequence was as follows: SIRT6 KO-01 (GTG TGA TTG GTA GAG AGG CAC GTG GAT), SIRT6 KO-02 (GCA ATA GCA TCA CAA ATT TCA CAA ATA), SIRT6 KO-03 (GTG CAT CTC AAT GGT GCA GTG CAT GTT). All animal procedures were performed in accordance with the institutional guidelines at Sichuan University (ethics record number: 20220301089). All mice were fed with a normal chow diet.

### 2.3 Platelet preparation

Blood was collected from the hearts of mice anesthetized with pentobarbital and added to 3.8% citrate anticoagulant. Platelet-rich plasma (PRP) was centrifuged at 250 *g* for 6 min and then at 600 g PRP for 5 min to precipitate platelets. Platelets were diluted with modified Tyrode buffer (in mM: 20 N-2-hydroxyethylpiperazine-N′-2-ethanesulfonic acid [HEPES], 137 NaCl, 13.8 NaHCO_3_, 2.5 KCl, 0.36 NaH_2_PO_4_, 5.5 glucose, pH 7.4).

### 2.4 Platelet aggregation

Washed platelets (2×10^8^/mL) from Wild-Type (WT) and SIRT6 KO mice was stimulated with thrombin (0.025 U/mL) under stirring conditions (1200 rpm) at 37°C using platelet aggregation analyzer (AG400) for 5 min. The experiment was performed after platelet count normalization (2×10^8^/mL). Platelets were incubated with UBCS039 (100 μmol/L) for 30 min.

### 2.5 Flow cytometry

Fluorophore-labelled antibodies were utilized for the detection of P-selectin expression (CD62P-PE) and the active form of αIIbβ3 integrin (JON/A binding) for 20 min in the dark at room temperature. Resting and washed platelets (1 × 10^7^/mL) activated by thrombin (0.01 U/mL) for 5 min were pretreated with UBCS039 (100 μM) for 30 min. Data were analyzed using FlowJo v10 software.

### 2.6 Platelet spreading

Platelets (2 × 10^7^/mL) were placed on fibrinogen-coated glass coverslips (100 μg/mL fibrinogen, 4°C overnight) at 37°C for 90 min. After washing with modified Tyrodes HEPES buffer, the platelets were fixed, permeabilized, stained with Alexa Fluor-546-labelled phalloidin, and viewed with fluorescence microscopy (Nikon-80i) using a ×100 oil objective.

### 2.7 Scanning electron microscopy analysis

The washed platelets were fixed in 3% glutaraldehyde 0.1 M sodium carbonate buffer (pH 7.4) and dehydrated with gradient ethanol. The sample was placed on a 2 mm glass slide, sprayed with gold and dried. A filter paper immersed in 10% FeCl_3_ was placed on the top of left carotid artery immediately. 5 min later, the filter paper was removed and the injured area was rinsed twice with PBS. After 45 min, the carotid artery was stripped and the thrombus was removed and fixed in 4% paraformaldehyde at room temperature. The specimens were observed under a JEM transmission electron microscope (EVO 10).

### 2.8 Clot retraction tests

Platelets (3 × 10^8^/mL) were supplemented with 0.5 mg/mL fibrinogen, and clot retraction was initiated with thrombin (1 U/mL) stimulation at 37 °C. Images were captured every 10 min.

### 2.9 FeCl_3_-induced thrombosis model

Exogenous calcein AM labeled platelets were injected into the tail vein. The mice were anesthetized with 1% pentobarbital, and then the common carotid artery was carefully exposed and kept moist with normal saline, and the filter paper was saturated with 5% ferric chloride (FeCl_3_) solution (5 × 3 mm) for 1 min. The formation of thrombus in the injured carotid artery was monitored with a Nikon A1 RMP^+^two-photon microscope.

### 2.10 Adoptive transfer experiments

After the application of R300, extracted WT and SIRT6^−/−^ mice platelets were injected into SIRT6^−/−^ mice and WT mice, respectively, through caudal veins. The infusion liquid was controlled within 100 μL to prevent death caused by excessive liquid. After the infusion, the mice were kept in cages and modeling was completed within 48 h.

### 2.11 Western blotting

Washed WT in the presence or absence of SBC115076 (100 μM) or SIRT6^−/−^ platelets (250 μL; 2 × 10^8^/mL) were stimulated with thrombin. RIPA lysis buffer was added to extract total protein from platelets. Proteins were separated by SDS-PAGE gel electrophoresis and transferred to PVDF membranes, which were incubated with primary antibodies SIRT6 (1:1000, HUABIO) and GAPDH (1:1000, Abcam). After incubation with the corresponding secondary antibodies, the protein bands were subject to chemiluminescence and quantitative analysis using the Odyssey Fc System (LI-COR Biosciences) and Image lab software.

### 2.12 H&E staining

The thrombus samples were fixed and embedded in paraffin. Thrombotic sections were stained with H&E. Use Axio Observer Z1 inverted fluorescence microscope to obtain images of immunofluorescence staining. The image was processed by Zen 2012 (blue version, version 2.3, Zeiss) software.

### 2.13 Statistical analysis

All data are expressed as mean ± SD. Data were evaluated with one-way ANOVA or two-way ANOVA. Student’s t*-*test was used for comparisons between 2 groups. The statistically significant difference was considered to be *p* < 0.05. GraphPad Prism 8.0 was used for statistical analysis.

## 3 Results

### 3.1 SIRT6 activation suppresses FeCl_3_-induced arterial thrombosis in mice

**FIGURE 1:**
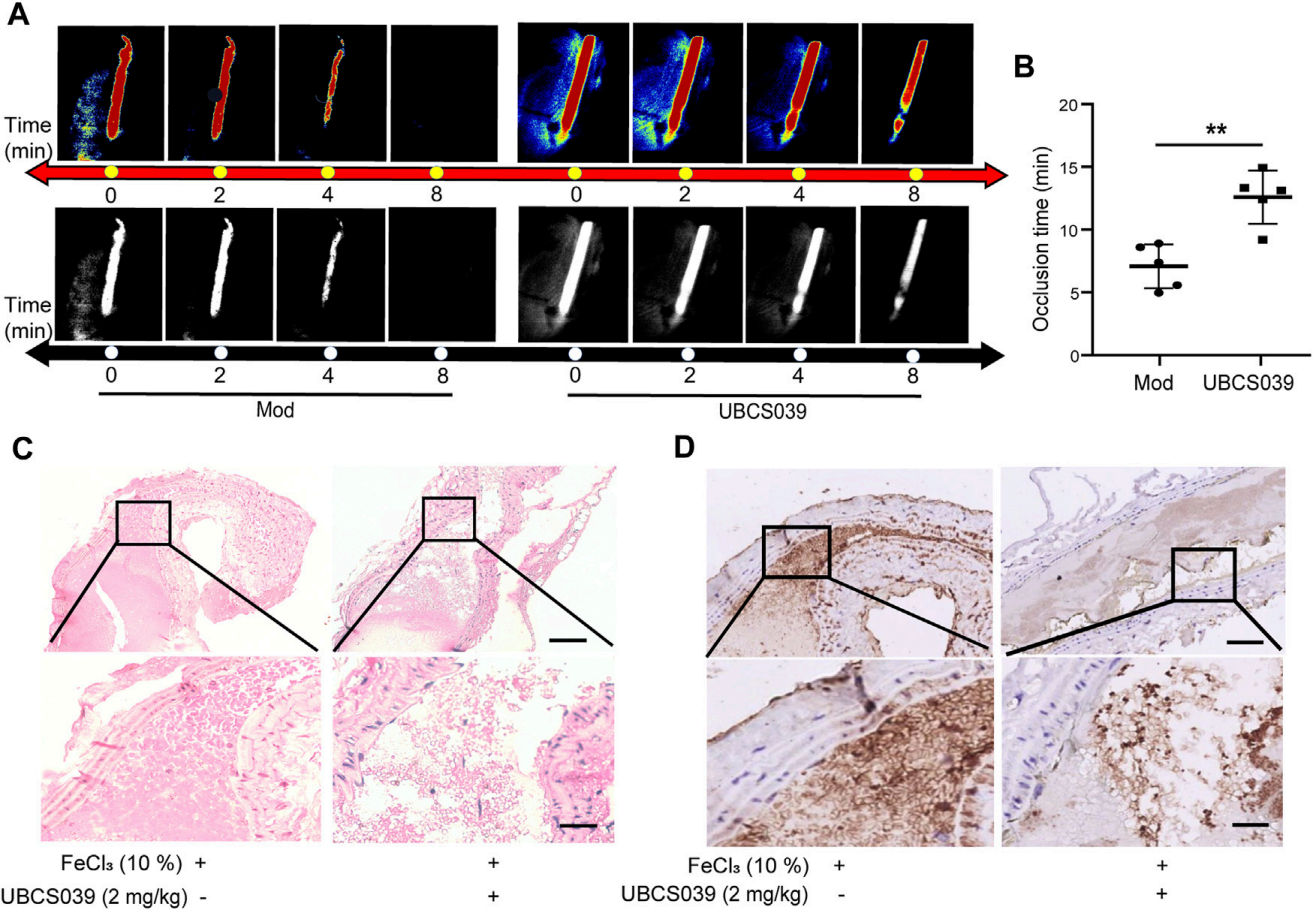
SIRT6 agonist treatment inhibits FeCl_3_-induced arterial thrombus formation in mice. **(A)** Representative images of blood flow through carotid arteries which had been damaged by 10% FeCl_3_ (Model, Mod). The blood flow was detected using the laser speckle imaging technique. The red part, which represented the blood flow signal, became weak and finally disappeared as time went on. **(B)** Quantification of vascular occlusion time. The results are shown as mean ± SD (n = 5), **p < 0.01. **(C)** Representative images of H&E staining of arterial thrombosis. **(D)** Representative immunohistological images of CD41-positive platelets (n = 3). Scale bar represents 50 μm (up). Bar represents 10 μm (down).

To investigate the role of SIRT6 in thrombosis, we first performed a FeCl_3_-induced arterial thrombosis model in C57BL/6 mice and examined the effect of SIRT6 activation on thrombosis by intraperitoneal injection of the specific SIRT6 agonist, UBCS039. Laser speckle imaging was employed to analyze the changes of arterial thrombus occlusion time. As shown in Figures 1A, B, UBCS039 treatment significantly prolonged the mean time to thrombotic occlusion. Furthermore, hematoxylin-eosin (H&E) staining revealed that the thrombus in UBCS039-treated mice were looser than in untreated mice (Figure 1C). Immunohistochemical staining of CD41-labeled platelets also showed a marked reduction in the number of platelets in the thrombus (Figure 1D). Taken together, these results suggest that SIRT6 may play a potential inhibitory role in thrombus formation.

### 3.2 Platelet SIRT6 negatively regulates multiple processes of thrombin-induced platelet activation

**FIGURE 2:**
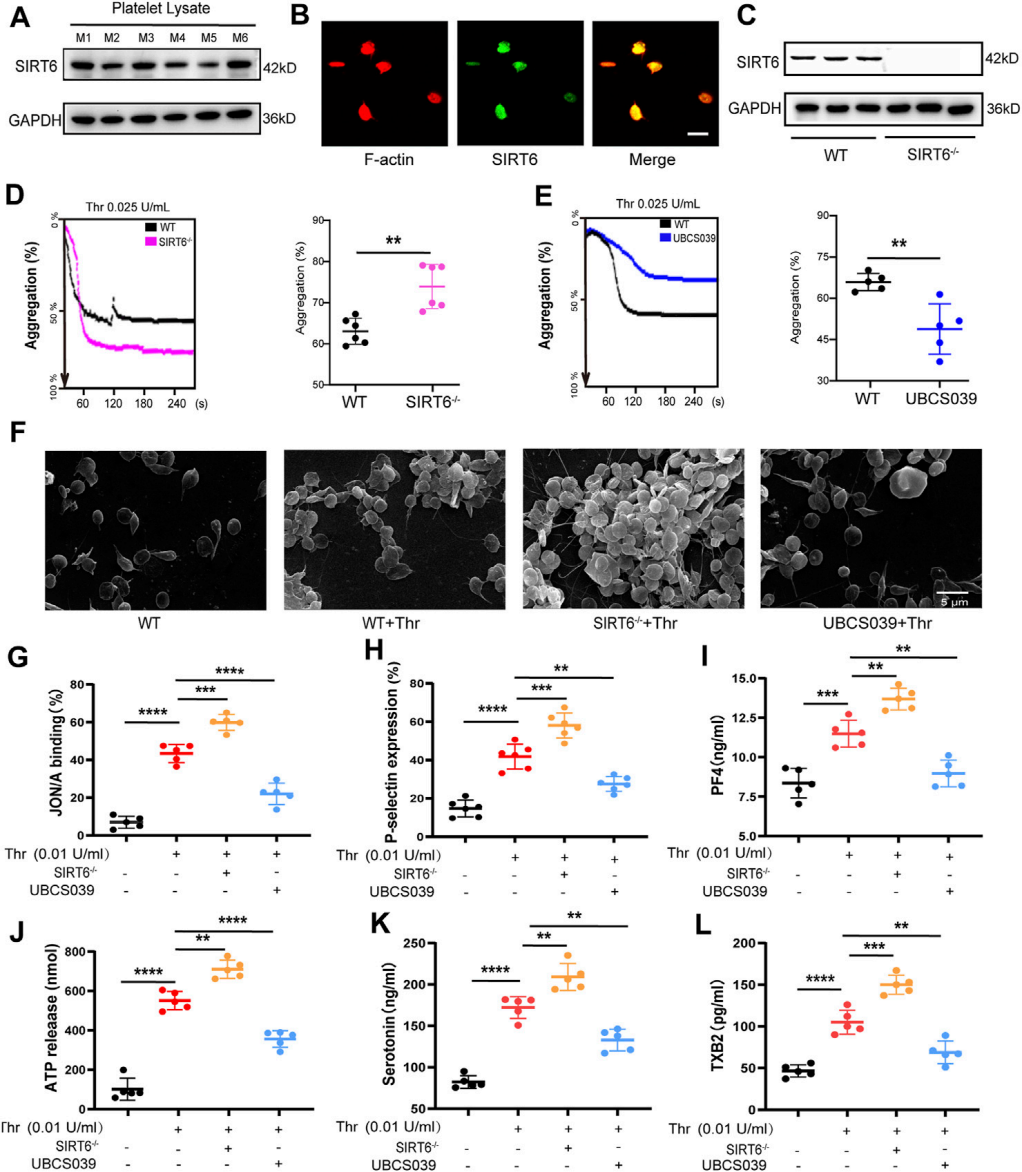
SIRT6 in platelets is essential for thrombin-induced platelet activation. **(A)** Each of the six lanes in the Western blot represents independent platelet lysate samples isolated from normal C57BL/6 mice, aiming to confirm the expression of SIRT6 protein in platelets. M1 to M6 correspond to six different mice. **(B)** Immunofluorescence images of the protein expression of SIRT6 in platelet, the scale represents 5 µm. **(C)** Analysis of SIRT6 expression in platelets from WT and SIRT6^−/−^ mice by immunoblot. **(D,E)** Effect of SIRT6 deficiency and pre-treatment with UBCS039 (100 μM) on the thrombin-induced aggregation of mouse washed platelets assayed using the AG400 semi-automaticplatelet aggregation analyzer, **p < 0.01. **(F)** Representative scanning electron micrographs of WT mice platelets treated with or without UBCS039 and SIRT6^−/−^ isolated platelets. **(G,H)** Flow cytometric analysis of PE-JON/A and FITC-CD62P binding to platelets stimulated with thrombin for 5 min at 37 °C, n = 5-6. **(I)** PF4 released from α-granule granules was measured by ELISA. **(J)** ATP and **(K)** serotonin released from dense granules were measured by microplate reader and ELISA. **(L)** TXB2 secretion in thrombin was measured by ELISA. The results are shown as mean ± SD (n = 5-6), **p < 0.01,***p < 0.001, ****p < 0.0001.

To explore whether SIRT6 in platelets is directly involved in the regulation of platelet homeostasis, we first characterized the presence of SIRT6 protein in mouse platelets by Western blotting and immunofluorescence (Figures 2A, B). Next, we isolated platelets from SIRT6 knockout (SIRT6^−/−^) mice and examined the effect of platelet SIRT6 deletion on platelet morphology and function. Western blotting analysis confirmed the absence of SIRT6 protein in platelets of SIRT6^−/−^ mice, wherase the expression of other sirtuins family members, including SIRT1 and SIRT2, was unaffected in SIRT6^−/−^ platelets (Figure 2C and Supplementary Figure S1). Further platelet aggregation assays and scanning electron microscopy analysis revelaed that platelets lacking SIRT6 significantly enhanced thrombin-induced platelet aggregation, whereas SIRT6 activation by UBCS039 pretreatment showed significant aggregation defects (Figures 2D–F and Supplementary Figure S2).

Next, we sought to explore how SIRT6 affects platelet aggregation by examining markers associated with core processes of platelet activation. We found that integrin αIIbβ3 activation (measured by JON/A binding), α-granules (P-selectin and PF4) release, dense (ATP and serotonin) granules release, and self-amplification of platelet activation (measured by thromboxane B2 production) were all potentiated in washed platelets form SIRT6^−/−^ mice stimulated with thrombin. In contrast, these thrombin-induced platelet activation processes were significantly inhibited in UBCS039-pretreated platelets (Figures 2G–L). Moreover, UBCS039 pretreatment in SIRT6^−/−^ platelets showed no inhibitory effect on thrombin-induced platelet integrin activation (JON/A binding) and granule release (P-selectin) (Supplementary Figure S2). Collectively, these data suggest that SIRT6 in platelets negatively regulates multiple processes of agonist-induced platelet activation.

### 3.3 Platelet SIRT6 inhibits platelet spreading and clot retraction

**FIGURE 3:**
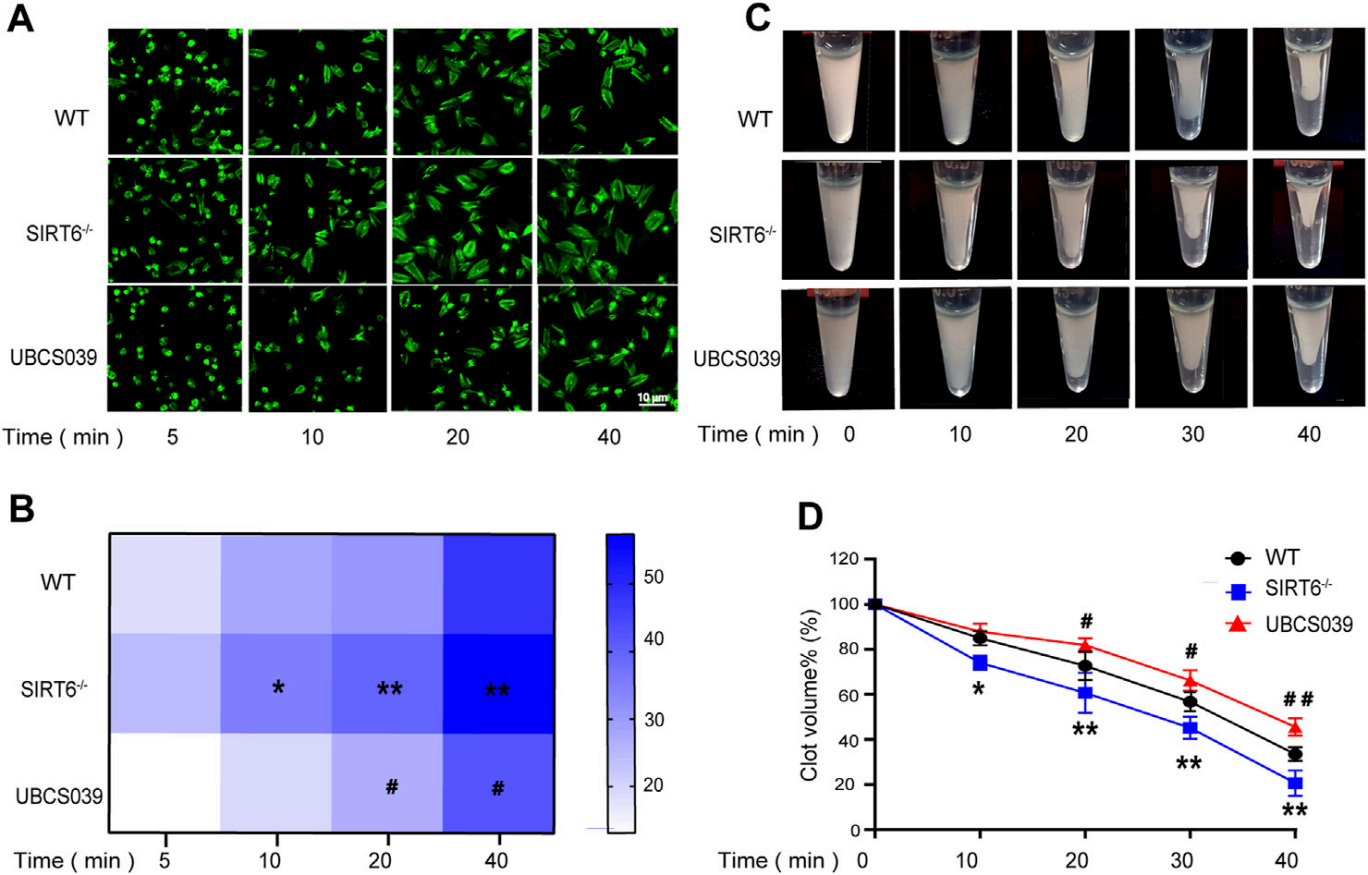
Effect of SIRT6 in platelets on platelet spreading and clot retraction. SIRT6^−/−^ platelet and WT platelets pretreated with or without UBCS039 spread on fibrinogen with thrombin (0.01 U/mL) stimulation at 37 °C for 5–40 min **(A,B)** The spreading area of single platelets was measured using ImageJ software. **(C,D)** Clot retraction was measured for 40 min in PRP with 150 μg/mL purified fibrinogen, after adding 0.1 U/mL of thrombin in plateles. Results were quantified and presented as mean ± SD, *p < 0.05, **p < 0.01.

Given that activation of integrin αIIbβ3-mediated outside-in signaling would lead to platelet spreading and clot retraction (Shattil et al., 1998; Dai et al., 2016; Huang et al., 2019), we thus examined the effect of SIRT6 depletion or activation on platelet spreading and clot retraction. As expected, SIRT6 deletion promoted thrombin-induced platelet spreading on fibrinogen (Figures 3A, B) and accelerated clot retraction in platelet suspension (Figures 3C, D), which was significantly inhibited by UBCS039 pretreatment. Together, these data further elucidate the negative regulation of endogenous SIRT6 on platelet activation.

### 3.4 SIRT6 in platelets attenuates thrombin-induced activation of MAPK signaling pathway

**FIGURE 4:**
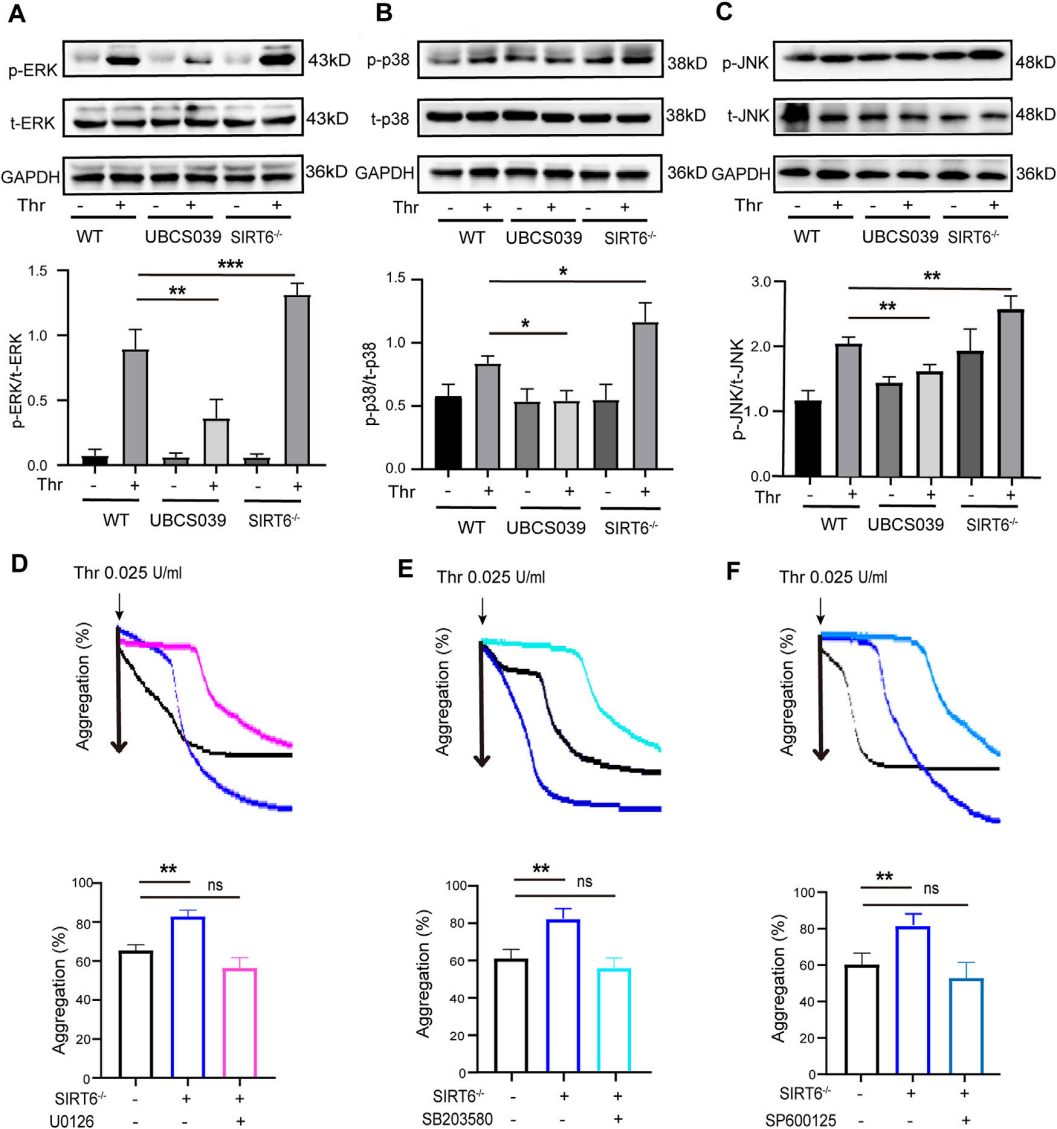
Effects of platelet SIRT6 on thrombin-induced activation of MAPK signaling. **(A–C)** Platelets were pretreated with UBCS039 (100 μM) for 30 min at room temperature before stimulation with thrombin (0.025 U/mL). The above panels show representative western blots for phospho-p38, total p38, phospho-ERK, total ERK, phospho-JNK, and total JNK. The down panels show densitometry analysis of immunoblots. The results are shown as mean ± SD (*n* = 4). Data were analyzed using one-way ANOVA followed by Bonferroni post hoc analysis. *p < 0.05, **p < 0.01, ***p < 0.001. **(D–F)** Representative aggregation tracings of platelets. Washed platelets were pretreated with ERK inhibitor U0126 (10 μM), p38 inhibitor SB203580 (10 μM) and JNK inhibitor SP600125 (10 μM) for 30 min, then treated with thrombin (0.025 U/mL). The results are shown as mean ± SD (*n* = 4). Ns = *p* > 0.05, **p < 0.01.

The MAPK signaling pathway plays a key role in mediating multiple processes of platelet activation (Kansra and Shukla, 2000; Adam et al., 2008; Lee et al., 2010). We thus sought to explore the regulatory effect of SIRT6 depletion or activation on the phosphorylation of MAPK kinases such as ERK, JNK and p38. Western blotting analysis showed that the phosphorylation levels of ERK (Thr202/Tyr204), p38 (Thr180/Tyr182) and JNK in SIRT6^−/−^ platelets were significantly enhanced compared with WT platelets after thrombin stimulation Conversely, UBCS039 pretreatment effectively abolished thrombin-induced phosphorylation of the aforementioned MAPK kinases (Figures 4A–C). To further confirm that MAPK signaling pathway is involved in mediating the inhibitory effect of SIRT6 on platelet activation, we pretreated washed platelets in WT or SIRT6−/− mice prior to thrombin stimulation with specific inhibitors of ERK (U0126), p38 (SB203580), or JNK (SP600125), respectively. As shown in Figures 4D, E, inhibition of MAPK signaling reversed the promoting effect of SIRT6 deficiency on platelet aggregation. Therefore, our data suggest that SIRT6 in platelets blocks the platelet activation process by inhibiting the MAPK signaling pathway.

### 3.5 PCSK9 mediates the inhibitory effect of SIRT6 on platelet activation

**FIGURE 5:**
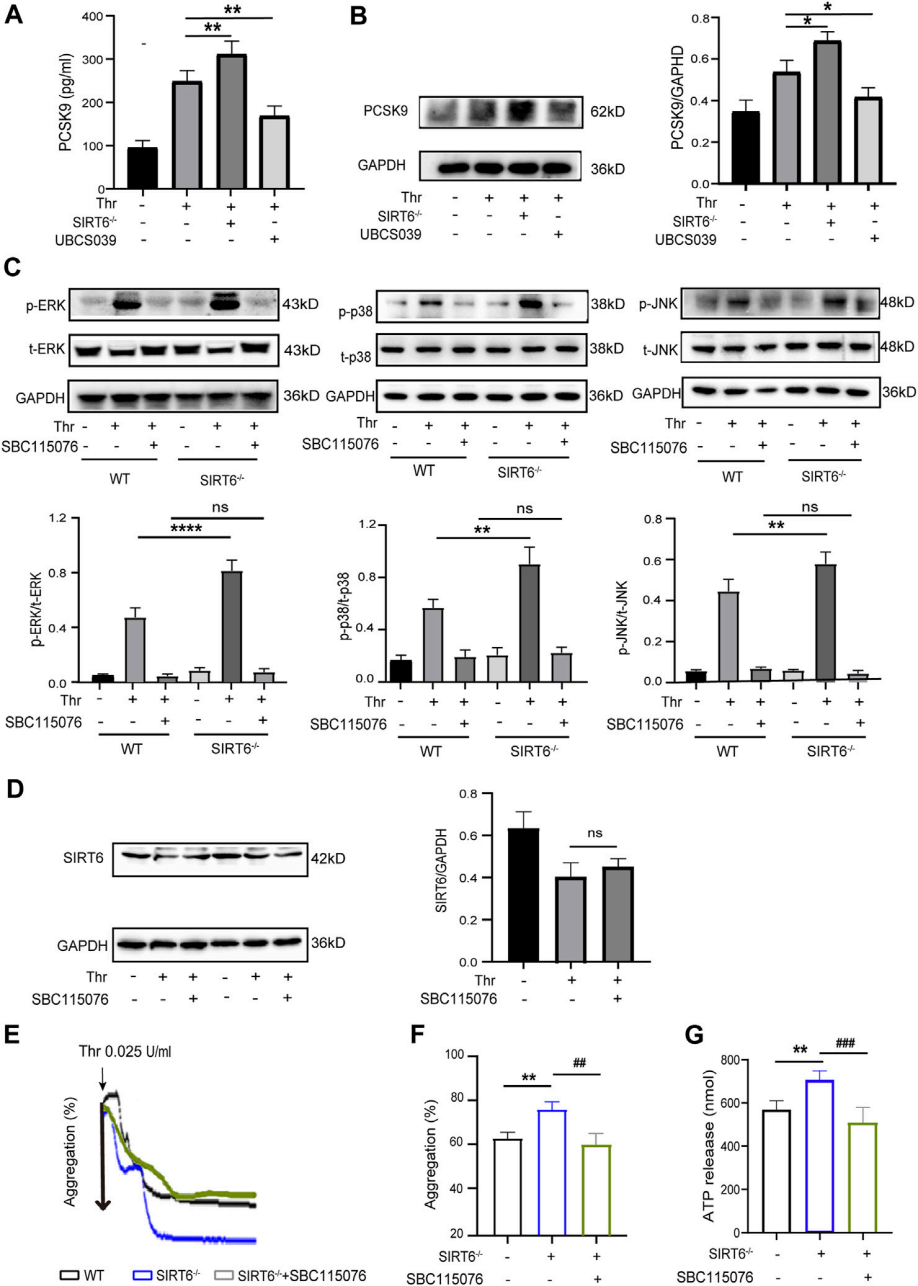
SIRT6 negatively regulates platelet PCSK9 secretion. **(A)** ELISA results showed that the absence of SIRT6 in platelets increased the release of PCSK9. **(B)** Western blot results showed that SIRT6 deficiency increased the expression of PCSK9. Results were quantified and presented as mean ± SD. **(C)** SIRT6^−/−^ platelets were pretreated with or without PCSK9 inhibitors SBC115076 (100 μM) for 30 min at room temperature before stimulation with thrombin (0.025 U/mL). The above panels show representative western blots for phospho-p38, total p38, phospho-ERK, total ERK, phospho-JNK, and total JNK. The down panels show densitometry analysis of immunoblots, **p < 0.01,***p < 0.001 vs. WT + Thr, ns = *p* > 0.05 vs. WT + Thr + SBC115076. **(D)** The expression of SIRT6 in thrombin (0.025 U/mL, 15 min) stimulated platelets preincubated with PCSK9 inhibitors SBC115076 (100 μM, 30 min) was detected by Western blot analysis. **(E,F)** The aggregation tracings of thrombin (0.025 U/mL) stimulated WT or SIRT6^−/−^ platelets treated without or with PCSK9 inhibitors SBC115076 (100 μM, 30 min). **(G)** ATP secretion in thrombin (0.025 U/mL) stimulated-platelets. The data presented as mean ± SD, and were analyzed using one-way ANOVA followed by Bonferroni *post hoc* analysis. **p < 0.01 vs. the WT group, ^##^ *p* < 0.01, ^###^ *p* < 0.001 vs. the SIRT6^−/−^ + SBC115076 group.

PCSK9 enhances platelet activation by binding to CD36 in platelets, while SIRT6 has been shown to interact with PCSK9 to inhibit its secretion (Cammisotto et al., 2020; Da Dalt et al., 2021; Qi et al., 2021). Therefore, we sought to investigate whether PCSK9 is involved in mediating the inhibition of platelet activation by SIRT6. We first examined the release of platelet-derived PCSK9 by ELISA assay. As shown in Figure 5A, the release of PCSK9 was further increased in SIRT6^−/−^ platelets after thrombin stimulation compared with WT platelets, whereas thrombin-induced release of PCSK9 was significantly abrogated in WT platelets pretreated with UBCS039. Likewise, Western blot analysis further verified that PCSK9 protein expression levels were increased in SIRT6^−/−^ platelets but decreased by UBCS039 preincubation (Figure 5B). These data suggest that PCSK9 may be a core executive molecule negatively regulated by SIRT6, which triggers platelet activation.

Next, to further explore whether the altered level of platelet-derived PCSK9 release mediates the inhibitory effect of SIRT6 on the MAPK signaling pathway, we measured changes in the phosphorylation levels of the above-mentioned MAPK kinases. Interestingly, pretreatment of WT platelets with a PCSK9 antagonist, SBC115076, did not affect the phosphorylation levels of ERK (Thr202/Tyr204), p38 (Thr180/Tyr182) and JNK, regardless of stimulation with thrombin. However, in SIRT6-deficient platelets, SBC115076 pretreatment effectively inhibited the activation of these MAPK kinases (Figure 5C). Furthermore, SBC115076 treatment had no effect on platelet SIRT6 protein levels (Figure 5D), suggesting that PCSK9 is a downstream effector molecule of SIRT6 and an upstream regulator of the MAKP signaling pathway. In addition, platelet function assays also confirmed that antagonizing PCSK9 abolished the differences in platelet aggregation and ATP release between WT and SIRT6^−/−^ mice. (Figures 5E–G). Taken together, our data suggest that SIRT6 regulates the release of PCSK9 from platelets, leading to the activation of its downstream MAPK signaling pathway and ultimately platelet activation.

### 3.6 SIRT6 depletion promotes FeCl_3_-induced arterial thrombosis in mice

**FIGURE 6:**
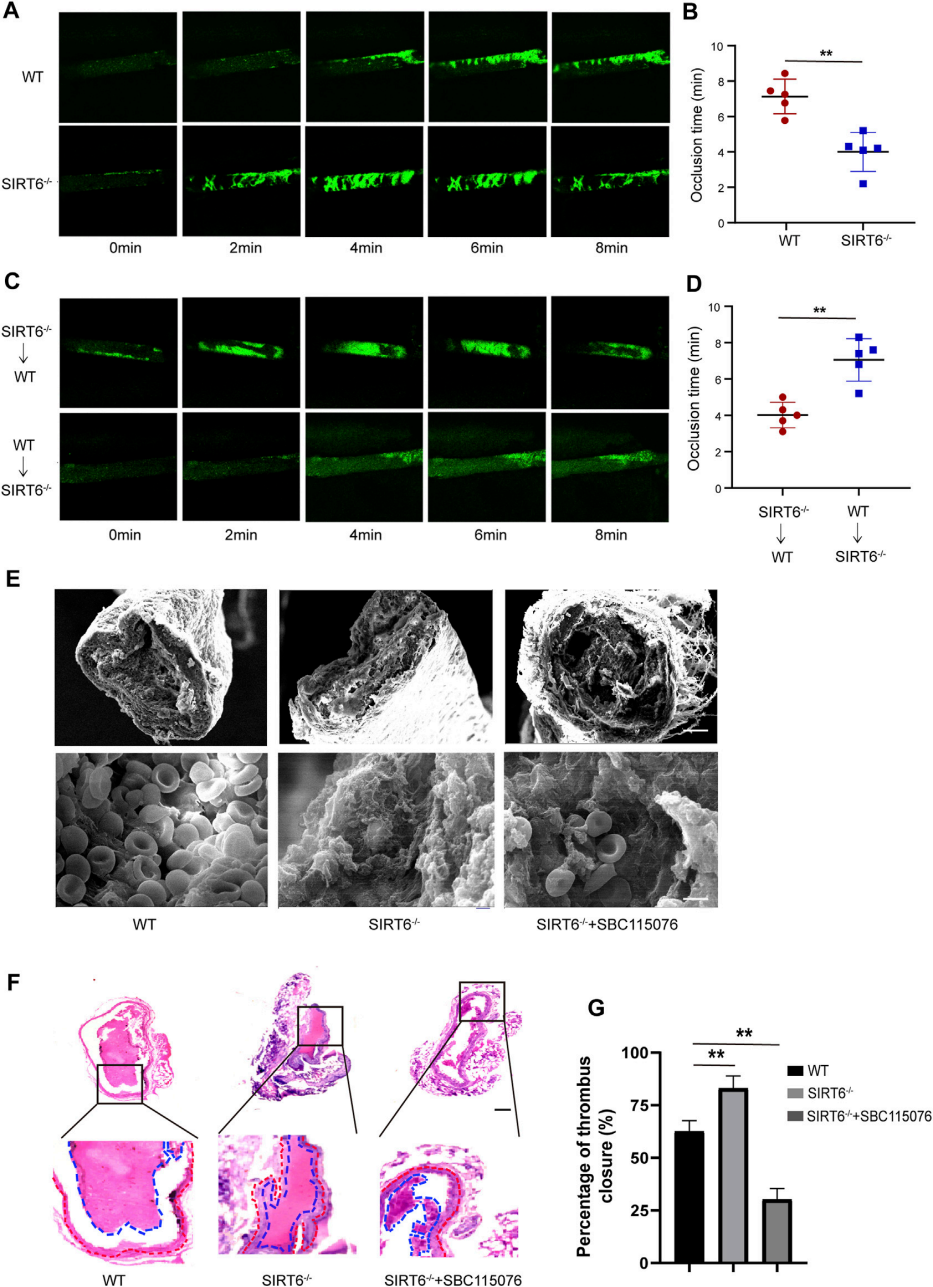
Platelet SIRT6 deletion promotes carotid artery thrombosis. **(A,B)** Time courses of FeCl_3_-induced vascular injury and thrombus formation in WT and SIRT6^−/−^ mice, recording of microscopic images every 2 min (n = 4). **(C,D)** WT or SIRT6^−/−^ mice were treated with specific anti-CD42b antibodies (R300 0.5 μg/g) to deplete platelets. Time courses of FeCl_3_-induced vascular injury and thrombus formation. **p < 0.01. **(E)** Scanning electron microscopy of SIRT6^−/−^ mice with or without SBC115076 (2 mg/kg). Scale bar represents 50 μm (up). Bar represents 2 μm (down). (n = 3). **(F)** H&E staining images in a carotid artery thrombus (n = 3). The results are expressed as mean ± SD. Bar represents 50 μm. **(G)** Percentage of thrombus closure in each group.

Next, we assessed the effect of SIRT6 depletion on thrombosis and hemostasis *in vivo*. As shown in Figures 6A, B, the time required for SIRT6^−/−^ mice to form a stable, occlusive thrombus in FeCl_3_-induced arterial thrombosis was significantly shorter than that of WT mice.

To further confirm the direct inhibitory effect of platelet SIRT6 on thrombus formation, platelet cross-exchange experiments were performed. Recipient WT mice or SIRT6^−/−^ mice were pretreated with anti-CD42b antibody to deplete endogenous platelets (Supplementary Figure S3), followed by intravenous infusion of fluorescently labeled SIRT6^−/−^ or WT platelets, respectively. As expected, SIRT6^−/−^ mice transfused with WT platelets displayed a significant prolongation of occlusion time compared with WT mice transfused with SIRT6^−/−^ platelets (Figures 6C, D). Moreover, scanning electron microscopy and histopathological examination showed that the structural morphology of the thrombus formed in SIRT6-deficient mice was more dense than that in WT mice, but was significantly improved after intraperitoneally infusion of PCSK9 antagonism (Figures 6E–G). Thus, these findings provide further *in vivo* evidence that SIRT6 in platelets ameliorate carotid thrombosis at least in part by inhibiting PCSK9 secretion.

## 4 Discussion

Platelets play a central role in the pathogenesis of arterial thrombosis, and the mechanisms regulating the adhesive functions of platelets are thus of pivotal importance for occlusive cardiovascular disease (Barnhart, 1978; Kroll and Schafer, 1989). Recent research provides some evidence for a link between the sirtuins family and thrombosis (Wu et al., 2022). In this study, we demonstrated for the first time that SIRT6 is expressed in platelets and negatively regulates platelet function, including integrin αIIbβ3 activation, granules release, and platelet adhesion. The protective effect of SIRT6 on platelet activation was attributed to the reduction of platelet-derived PCSK9 secretion, and the subsequent inhibition of its downstream MAPK signaling pathway.

The protective role of the SIRT family, particularly SIRT1 and SIRT6, in cardiovascular disease has been extensively investigated over the past few decades (D'Onofrio et al., 2016; D'Onofrio et al., 2018). Notably, the activation of SIRT1 hinders abnormal vascular smooth muscle cell proliferation and protects endothelial cells from damage, thus impeding the progression of atherosclerosis (Gorenne et al., 2013). SIRT6 is closely linked to aging-related cardiovascular diseases due to its involvement in various processes, including oxidative stress, inflammatory responses, autophagy, genome integrity, and telomere homeostasis (Winnik et al., 2015; Grootaert et al., 2021; Bian et al., 2022). Recently, a study revealed that targeted deletion of SIRT6 in the endothelium of mice promotes arterial thrombosis by increasing TF expression and activating pro-inflammatory signaling (Gaul et al., 2023), highlighting the importance of SIRT6 in regulating thrombotic processes. Indeed, our investigation demonstrated robust SIRT6 expression in mouse washed platelets. Manipulation of SIRT6 in platelets, either through genetic knockout or agonist activation, significantly impacted platelet function, affecting integrin αIIbβ3 activation, granule release, and platelet adhesion. These findings underscore the critical importance of platelet endogenous SIRT6 in maintaining platelet homeostasis. Moreover, by using a FeCl_3_-induced arterial thrombosis mouse model, we confirmed the protective effect of SIRT6 in the reduction of thrombus occlusion time and thrombus area. In view of a recent study showing that SIRT6 in vascular endothelial cells has a protective effect on arterial thrombosis (Gaul et al., 2023), we carried out platelet cross-exchange experiments and further confirmed that endogenous SIRT6 in platelets plays a direct negative regulatory role in mediating thrombus formation.

Numerous studies have demonstrated the presence and activation of specific MAPKs, including ERK, p38, and JNK, in platelets in response to various agonists (Lee et al., 2010; Manne et al., 2022). Activation of ERK in platelets has been shown to be critical for collagen-induced platelet secretion and aggregation (Oury et al., 2002), and treatment with an inhibitor of its upstream MEK1/2 inhibited ERK activation and prolonged occlusion time in mouse arteriole and venule thrombosis (Dai et al., 2022). In addition, p38 activation in collagen-stimulated platelets resulted in an increase in platelet adhesion and spreading and thromboxane A2 formation (Saklatvala et al., 1996). Deletion of JNK in platelets also exhibited impaired platelet aggregation and granule release in response to agonist stimulation and suppressed thrombus formation in a mouse model induced by photochemical injury of cecal vessels (Adam et al., 2010). Likewise, in this study we showed that both ERK, p38, and JNK were involved in SIRT6-mediated inhibition in thrombin-induced platelet activation. We demonstrated that platelet SIRT6 deletion increased the phosphorylation of ERK, p38, and JNK, while inhibition of MAPK signaling reversed the promoting effect of SIRT6 deficiency on platelet aggregation, indicating a specific regulatory role of platelet endogenous SIRT6 on MAPK signaling pathways during platelet activation.

A recent study showed that PCSK9 in plasma directly enhances platelet activation and *in vivo* thrombosis by binding to platelet CD36 (Qi et al., 2021). Herein, we discovered that SIRT6 negatively regulates platelet PCSK9 expression and secretion. Interestingly, the use of a specific PCSK9 inhibitor in our experiments markedly nullified the enhancement effect of SIRT6 deficiency on platelet activation and thrombus formation. This inhibition was primarily attributed to the dampening of phosphorylation levels of ERK (Thr202/Tyr204), p38 (Thr180/Tyr182), and JNK. These findings, coupled with existing literature, underscore the intricate involvement of SIRT6 in platelet function. Notably, SIRT6 has been previously documented to impede the transcriptional expression of PCSK9 by fostering histone demethylation in the promoter region (Tao et al., 2013). However, the mechanism through which SIRT6 influences the expression and secretion of PCSK9 in anucleated platelets remains elusive. Notably, recent studies have unveiled that SIRT1, a member of the Sirtuin family, can directly bind to the PCSK9 protein, leading to its deacetylation at specific sites (Lys243, Lys421, and Lys506) (Velagapudi et al., 2023). This action inhibits PCSK9 secretion and activity. Moreover, SIRT6 has been reported to directly interact with PCSK9 (Wang et al., 2023). A plausible explanation for the regulatory mechanism of SIRT6 on PCSK9 protein levels in platelets could involve the direct interaction of SIRT6 with PCSK9, promoting its deacetylation and thereby regulating its stability. While these insights broaden our understanding, further experiments are warranted to confirm the intricate regulatory mechanisms and shed light on the precise role of SIRT6 in modulating PCSK9 in platelets.

## 5 Conclusion

In summary, our data demonstrate a negatively regulatory role for platelet endogenous SIRT6 in platelet activation and thrombus formation. SIRT6 inhibits thrombin-induced platelet activation by suppressing platelet-derived PCSK9 secretion and subsequent MAPK signaling transduction. These findings provides valuable mechanistic insights into the intricate interplay between endogenous SIRT6 and platelet function, shedding light on potential therapeutic avenues for thrombotic disorders.

## Data Availability

The original contributions presented in the study are included in the article/Supplementary Material, further inquiries can be directed to the corresponding author.
