# Supplementary material for: Endogenous SIRT6 in platelets negatively regulates platelet activation and thrombosis
Source: Front Pharmacol. 2023 Dec 18;14:1268708. doi: 10.3389/fphar.2023.1268708 (PMC10766690; doi:10.3389/fphar.2023.1268708)
Supplement: Supplementary file 1 [file Table1.DOCX]

Supplementary Material

Endogenous SIRT6 in Platelets Negatively Regulates Platelet Activation and Thrombosis

Yanli Liu^1^†, Tao Wang^1^†, Qilong Zhou^1^†, Guang Xin^1^, Hai Niu^1^, Fan Li^1^, Yilan Wang^1^, Shiyi Li^1^, Yuman Dong^1^, Kun Zhang^1^, Lijuan Feng^1^, Wei Fu^1^, Boli Zhang^2^, Wen Huang^1*^

*** Correspondence:** Wen Huang huangwen@scu.edu.cn

## Supplementary Figures


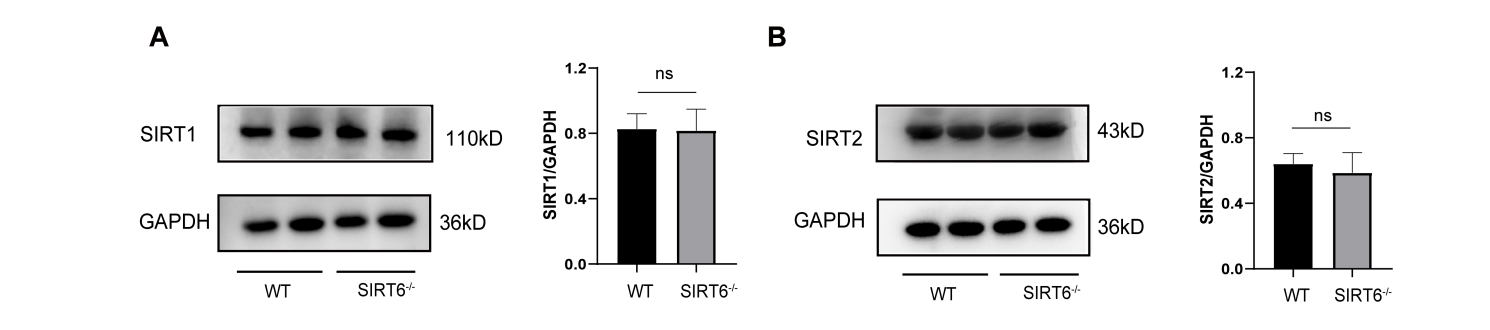


**Supplementary Figure 1. The expression of SIRT1 and SIRT2 in SIRT6^-/-^ platelets.** Western Blot results showed that SIRT6^-/-^ did not affect the expression of SIRT1and SIRT2. The results are quantified and expressed as mean ± SD, n=3. ns = *p* > 0.05.


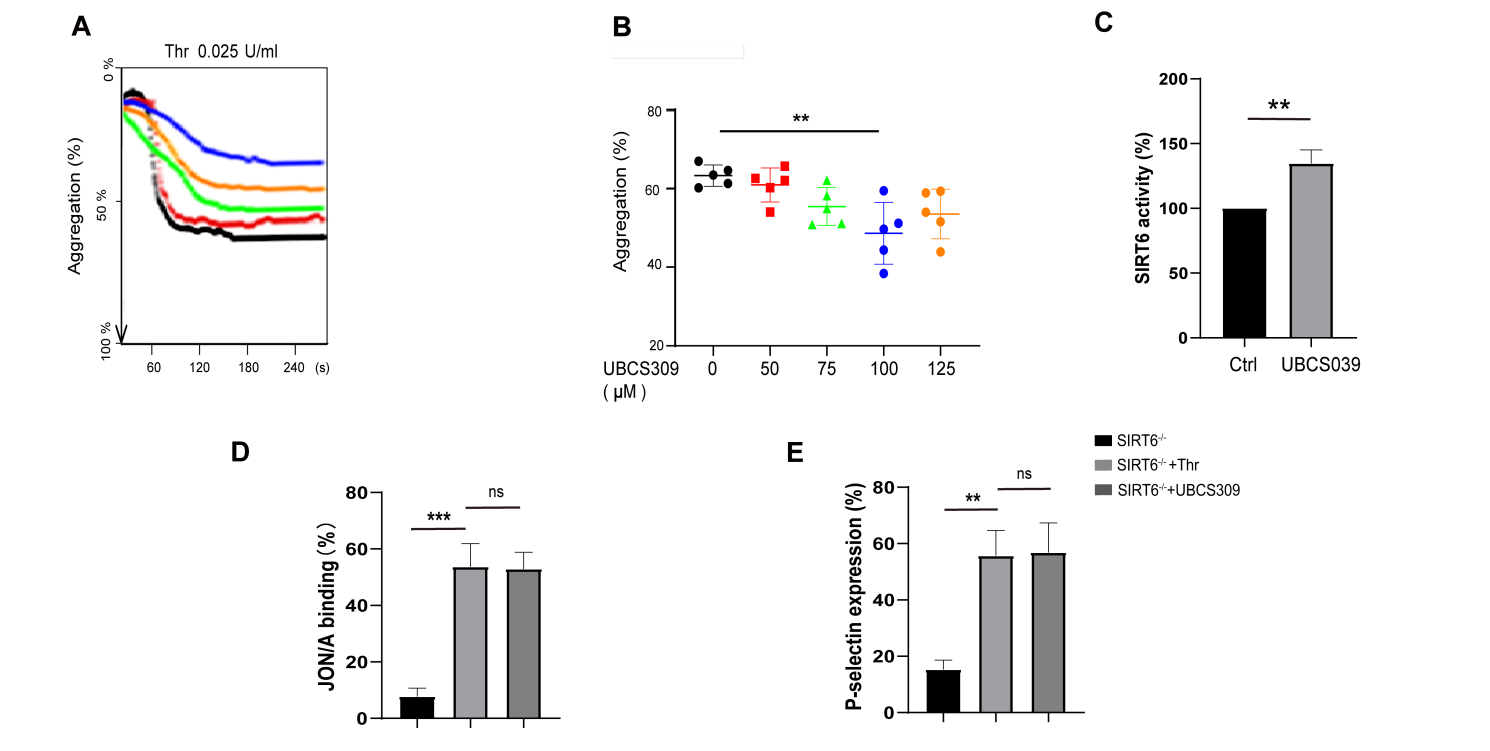


**Supplementary Figure 2. UBCS039 specifically inhibits platelet activation. (A-B)** Effect of platelets pre-treatment with different concentrations of UBCS039 on the thrombin-induced aggregation of mouse washed platelets assayed using the AG400 semi-automatic platelet aggregation analyzer, n=5. **(C)** ELISA determination of UBCS039 for platelet SIRT6 activity in vitro. The data presented as mean ± SD, n = 3. **(D-F)** Flow cytometric analysis of PE-JON/A and FITC-CD62P binding to platelets stimulated with thrombin for 5 min at 37 °C. The results are shown as mean ± SD, n=3. ns = *p* > 0.05, ***p* < 0.01.


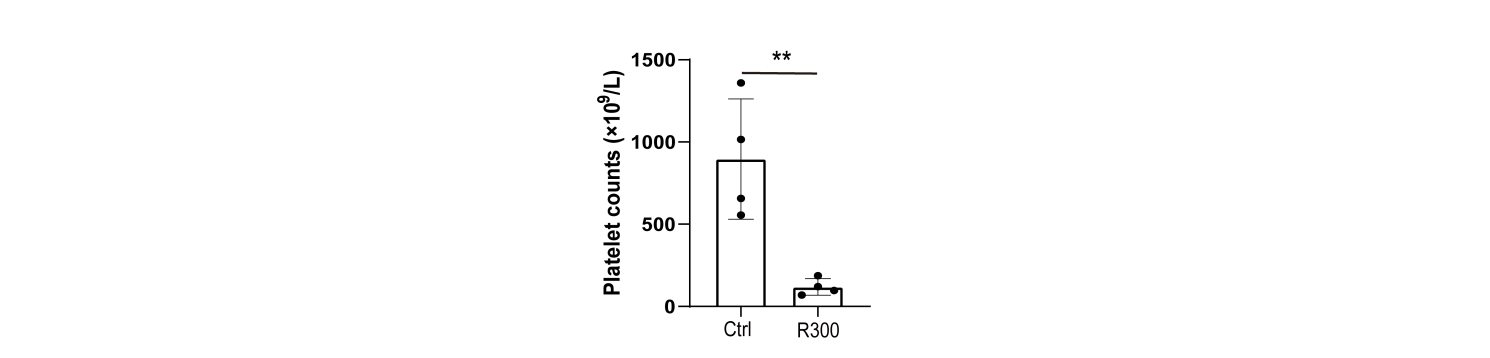


**Supplementary Figure 3. Mouse platelets depletion.** Mice were pretreated with anti-CD42b antibodies (R300). Thirty minutes after antibody treatment, blood was drawn and the number of blood platelets was counted using an automated hematology analyzer. The bar graph represents the mean, n=4. ***p* < 0.01.
